# 4-Chloro-*N*-(2,3-dimethyl­phen­yl)benzamide

**DOI:** 10.1107/S1600536811053256

**Published:** 2011-12-21

**Authors:** Vinola Z. Rodrigues, Jiří Kameníček,, B. Thimme Gowda, Jozef Kožíšek

**Affiliations:** a Department of Chemistry, Mangalore University, Mangalagangotri 574 199, Mangalore, India; b Department of Inorganic Chemistry, Palacký University, 17. listopadu 12, 771 46 Olomouc, Czech Republic; c Institute of Physical Chemistry and Chemical Physics, Slovak University of Technology, Radlinského 9, SK-812 37 Bratislava, Slovak Republic

## Abstract

In the title compound, C_15_H_14_ClNO, the *ortho*- and *meta*-methyl substituents in the aniline ring are *anti* to the N—H bond. The dihedral angle between the benzoyl and aniline benzene rings is 95.0 (1)°. N—H⋯O hydrogen bonds and C—H⋯π inter­actions link the mol­ecules in the crystal structure.

## Related literature

For the preparation of the title compound, see: Gowda *et al.* (1996 ▶, 2001 ▶). For our studies on the effects of substituents on the structures and other aspects of *N*-(ar­yl)-amides, see: Bowes *et al.* (2003 ▶); Gowda *et al.* (2001 ▶); Rodrigues *et al.* (2011 ▶), on *N*-(ar­yl)-methane­sulfonamides, see: Jayalakshmi & Gowda (2004 ▶), on *N*-(ar­yl)-aryl­sulfonamides, see: Gowda *et al.* (2005 ▶) and on *N*-chloro­aryl­amides, see: Gowda *et al.* (1996 ▶).
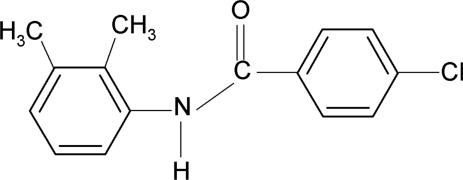


## Experimental

### 

#### Crystal data

C_15_H_14_ClNO
*M* *_r_* = 259.72
Monoclinic, 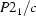

*a* = 8.1082 (8) Å
*b* = 19.5189 (17) Å
*c* = 9.2943 (9) Å
β = 111.957 (11)°
*V* = 1364.3 (2) Å^3^
*Z* = 4
Mo *K*α radiation
μ = 0.27 mm^−1^
*T* = 293 K
0.90 × 0.15 × 0.09 mm

#### Data collection

Oxford Diffraction Xcalibur Ruby Gemini diffractometer
Absorption correction: analytical [*CrysAlis RED* (Oxford Diffraction, 2009 ▶), based on expressions derived by Clark & Reid (1995 ▶)] *T*_min_ = 0.953, *T*_max_ = 0.976
22574 measured reflections
2793 independent reflections
1923 reflections with *I* > 2σ(*I*)
*R*_int_ = 0.058

#### Refinement

*R*[*F* ^2^ > 2σ(*F* ^2^)] = 0.049
*wR*(*F* ^2^) = 0.145
*S* = 1.04
2793 reflections
165 parameters
H-atom parameters constrained
Δρ_max_ = 0.40 e Å^−3^
Δρ_min_ = −0.30 e Å^−3^

### 

Data collection: *CrysAlis CCD* (Oxford Diffraction, 2009 ▶); cell refinement: *CrysAlis CCD*; data reduction: *CrysAlis RED* (Oxford Diffraction, 2009 ▶); program(s) used to solve structure: *SHELXS97* (Sheldrick, 2008 ▶); program(s) used to refine structure: *SHELXL97* (Sheldrick, 2008 ▶); molecular graphics: *DIAMOND* (Brandenburg, 2002 ▶); software used to prepare material for publication: *enCIFer* (Allen *et al.*, 2004 ▶).

## Supplementary Material

Crystal structure: contains datablock(s) I, global. DOI: 10.1107/S1600536811053256/bq2325sup1.cif

Structure factors: contains datablock(s) I. DOI: 10.1107/S1600536811053256/bq2325Isup2.hkl

Supplementary material file. DOI: 10.1107/S1600536811053256/bq2325Isup3.cml

Additional supplementary materials:  crystallographic information; 3D view; checkCIF report

## Figures and Tables

**Table 1:** Hydrogen-bond geometry (Å, °) *Cg*1 and *Cg*2 are the centroids of C2–C7 and C8–C13 rings, respectively.

*D*—H⋯*A*	*D*—H	H⋯*A*	*D*⋯*A*	*D*—H⋯*A*
N1—H1⋯O1^i^	0.86	2.18	2.904 (2)	141
C14—H14*A*⋯*Cg*1^ii^	0.96	2.94	3.653 (2)	132
C7—H7⋯*Cg*2^i^	0.93	2.76	3.612 (2)	154

Symmetry codes: (i) 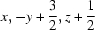; (ii) 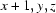.

**Fig. 1.**
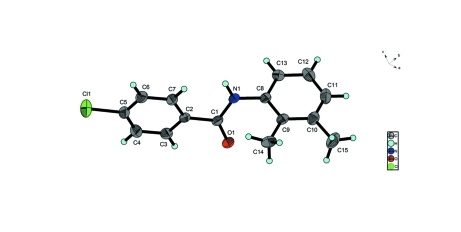
Molecular structure of the title compound showing the atom labeling scheme. Displacement ellipsoids are drawn at the 50% probability level.

**Fig. 2.**
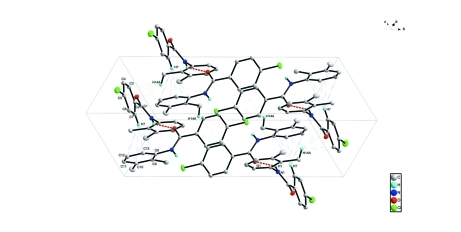
Part of the crystal structure of the title compound generated by N—H···O hydrogen bonds which are shown by dashed lines. C-H···π interactions are shown by thin dashed lines. H atoms not involved in intermolecular bonding have been omitted.

## supplementary crystallographic information

### Comment

The structural aspects of *N*-aryl amides are of interest due to their chemical and biological importance. As part of our studies on the substituent effects on the structures and other aspects of *N*-(aryl)-amides (Bowes *et al.*, 2003; Gowda *et al.*, 2001; Rodrigues *et al.*, 2011), *N*-(aryl)-methanesulfonamides (Jayalakshmi & Gowda, 2004), *N*-(aryl)-arylsulfonamides (Gowda *et al.*, 2005) and *N*-chloro-arylsulfonamides (Gowda *et al.*, 1996), in the present work, crystal structure of 4-chloro-*N*-(2,3-dimethylphenyl)benzamide (I) has been determined (Fig.1). In (I), the *ortho*- and *meta*-methyl substituents in the anilino ring are positioned *anti* to the N–H bond, similar to that observed in 4-methyl-*N*-(2,3-dimethylphenyl)benzamide (II) (Rodrigues *et al.*, 2011).

The central amide group –NHCO– is tilted to the anilino ring with the C9—C8—N1—C1 and C13—C8—N1—C1 torsion angles of -64.2 (3)° and 117.4 (2)°, compared to the corresponding values of -63.4 (2)° and 118.1 (1)° in (II). The C3—C2—C1—N1 and C7—C2—C1—N1 torsion angles are 158.0 (2)° and -22.5 (3)°, respectively, in contrast to the corresponding angles of 156.8 (1)° and -24.4 (2)° in (II). The C3—C2—C1—O1 and C7—C2—C1—O1 torsion angles are -21.9 (3)° and 157.6 (2)°, respectively, compared to the values of -23.2 (2)° and 155.6 (1)° in (II). The planes of two benzene rings are tilted relative to each other by 95.0 (1)°.

There are two clear C—H···pi interactions in the structure, C7—H7···*Cg*2 (Cg2 is the centroid of C8—C13 ring) and C14—H14A···*Cg*1 (*Cg*1 is the centroid of C2—C7 ring).

The packing of molecules linked by N—H···O hydrogen bonds is shown in Fig. 2.

### Experimental

The title compound was prepared according to the method described by Gowda *et al.* (1996, 2001). The purity of the compound was checked by determining its melting point. It was characterized by recording its infrared and NMR spectra.

Rod-like colourless single crystals of the title compound were obtained by slow evaporation from an ethanol solution of the compound (0.5 g in about 30 ml of ethanol) at room temperature.

### Refinement

All H atoms were visible in difference maps and then treated as riding atoms with C–H distances of 0.93 Å (C-aromatic), 0.96 Å (*C*-methyl) and N—H = 0.86 Å. The *U*_iso_(H) values were set at 1.2 *U*_eq_(C-aromatic, N) and 1.5 *U*_eq_(*C*-methyl).

### Crystal data

**Table:**

C_15_H_14_ClNO	*F*(000) = 544
*M**_r_* = 259.72	*D*_x_ = 1.265 Mg m^−^^3^
Monoclinic, *P*2_1_/*c*	Mo *K*α radiation, λ = 0.71073 Å
Hall symbol: -P 2ybc	Cell parameters from 3994 reflections
*a* = 8.1082 (8) Å	θ = 3.4–26.4°
*b* = 19.5189 (17) Å	µ = 0.27 mm^−^^1^
*c* = 9.2943 (9) Å	*T* = 293 K
β = 111.957 (11)°	Rod, colorless
*V* = 1364.3 (2) Å^3^	0.90 × 0.15 × 0.09 mm
*Z* = 4	

### Data collection

**Table:**

Oxford Diffraction Xcalibur Ruby Gemini diffractometer	2793 independent reflections
Radiation source: Enhance (Mo) X-ray Source	1923 reflections with *I* > 2σ(*I*)
graphite	*R*_int_ = 0.058
Detector resolution: 10.4340 pixels mm^-1^	θ_max_ = 26.4°, θ_min_ = 3.4°
ω scans	*h* = −10→10
Absorption correction: analytical [*CrysAlis RED* (Oxford Diffraction, 2009), based on expressions derived by Clark & Reid (1995)]	*k* = −24→24
*T*_min_ = 0.953, *T*_max_ = 0.976	*l* = −11→11
22574 measured reflections	

### Refinement

**Table:**

Refinement on *F*^2^	Primary atom site location: structure-invariant direct methods
Least-squares matrix: full	Secondary atom site location: difference Fourier map
*R*[*F*^2^ > 2σ(*F*^2^)] = 0.049	Hydrogen site location: inferred from neighbouring sites
*wR*(*F*^2^) = 0.145	H-atom parameters constrained
*S* = 1.04	*w* = 1/[σ^2^(*F*_o_^2^) + (0.0671*P*)^2^ + 0.3246*P*] where *P* = (*F*_o_^2^ + 2*F*_c_^2^)/3
2793 reflections	(Δ/σ)_max_ < 0.001
165 parameters	Δρ_max_ = 0.40 e Å^−^^3^
0 restraints	Δρ_min_ = −0.30 e Å^−^^3^

### Special details

**Table:**

Experimental. CrysAlis RED (Oxford Diffraction, 2009) Analytical numeric absorption correction using a multifaceted crystal model based on expressions derived (Clark & Reid, 1995).
Geometry. All e.s.d.'s (except the e.s.d. in the dihedral angle between two l.s. planes) are estimated using the full covariance matrix. The cell e.s.d.'s are taken into account individually in the estimation of e.s.d.'s in distances, angles and torsion angles; correlations between e.s.d.'s in cell parameters are only used when they are defined by crystal symmetry. An approximate (isotropic) treatment of cell e.s.d.'s is used for estimating e.s.d.'s involving l.s. planes.
Refinement. Refinement of *F*^2^ against ALL reflections. The weighted *R*-factor *wR* and goodness of fit *S* are based on *F*^2^, conventional *R*-factors *R* are based on *F*, with *F* set to zero for negative *F*^2^. The threshold expression of *F*^2^ > σ(*F*^2^) is used only for calculating *R*-factors(gt) *etc*. and is not relevant to the choice of reflections for refinement. *R*-factors based on *F*^2^ are statistically about twice as large as those based on *F*, and *R*- factors based on ALL data will be even larger.

### Fractional atomic coordinates and isotropic or equivalent isotropic displacement parameters (Å^2^)

**Table:**

	*x*	*y*	*z*	*U*_iso_*/*U*_eq_	
C1	0.7781 (3)	0.70473 (10)	0.5747 (2)	0.0420 (5)	
C2	0.6708 (3)	0.65266 (10)	0.6192 (2)	0.0412 (5)	
C3	0.5249 (3)	0.62384 (11)	0.5034 (2)	0.0512 (6)	
H3	0.4980	0.6366	0.4007	0.061*	
C4	0.4194 (3)	0.57674 (12)	0.5382 (3)	0.0569 (6)	
H4	0.3211	0.5579	0.4600	0.068*	
C5	0.4614 (3)	0.55780 (11)	0.6907 (3)	0.0520 (6)	
C6	0.6067 (3)	0.58405 (11)	0.8067 (3)	0.0527 (6)	
H6	0.6348	0.5700	0.9087	0.063*	
C7	0.7116 (3)	0.63166 (11)	0.7710 (2)	0.0477 (5)	
H7	0.8106	0.6498	0.8496	0.057*	
C8	0.9834 (3)	0.80088 (10)	0.6627 (2)	0.0409 (5)	
C9	1.1274 (3)	0.78514 (11)	0.6215 (2)	0.0442 (5)	
C10	1.2233 (3)	0.83941 (13)	0.5917 (3)	0.0555 (6)	
C11	1.1751 (3)	0.90633 (14)	0.6085 (3)	0.0653 (7)	
H11	1.2375	0.9423	0.5867	0.078*	
C12	1.0378 (3)	0.92083 (12)	0.6563 (3)	0.0605 (6)	
H12	1.0103	0.9660	0.6697	0.073*	
C13	0.9411 (3)	0.86789 (11)	0.6842 (2)	0.0488 (5)	
H13	0.8483	0.8771	0.7171	0.059*	
C14	1.1822 (3)	0.71233 (12)	0.6117 (3)	0.0608 (6)	
H14A	1.3083	0.7079	0.6662	0.073*	
H14B	1.1521	0.7000	0.5049	0.073*	
H14C	1.1214	0.6825	0.6576	0.073*	
C15	1.3817 (4)	0.82577 (19)	0.5477 (3)	0.0854 (9)	
H15A	1.4244	0.8683	0.5228	0.103*	
H15B	1.3471	0.7959	0.4592	0.103*	
H15C	1.4742	0.8045	0.6332	0.103*	
N1	0.8765 (2)	0.74774 (8)	0.68807 (18)	0.0440 (4)	
H1	0.8747	0.7429	0.7794	0.053*	
O1	0.7749 (2)	0.70745 (8)	0.44138 (16)	0.0549 (4)	
Cl1	0.32708 (12)	0.49969 (4)	0.73735 (11)	0.0947 (3)	

### Atomic displacement parameters (Å^2^)

**Table:**

	*U*^11^	*U*^22^	*U*^33^	*U*^12^	*U*^13^	*U*^23^
C1	0.0504 (12)	0.0442 (11)	0.0388 (11)	0.0029 (9)	0.0251 (10)	0.0015 (8)
C2	0.0484 (12)	0.0416 (11)	0.0401 (11)	0.0007 (9)	0.0240 (9)	−0.0021 (8)
C3	0.0616 (14)	0.0523 (13)	0.0416 (12)	−0.0033 (11)	0.0214 (11)	−0.0019 (9)
C4	0.0549 (14)	0.0555 (14)	0.0572 (14)	−0.0105 (11)	0.0172 (11)	−0.0070 (11)
C5	0.0592 (14)	0.0420 (12)	0.0643 (14)	−0.0042 (10)	0.0338 (12)	0.0003 (10)
C6	0.0682 (15)	0.0497 (13)	0.0467 (12)	0.0001 (11)	0.0287 (12)	0.0061 (10)
C7	0.0559 (13)	0.0501 (13)	0.0402 (11)	−0.0053 (10)	0.0216 (10)	0.0008 (9)
C8	0.0455 (11)	0.0463 (11)	0.0326 (10)	0.0001 (9)	0.0166 (9)	0.0014 (8)
C9	0.0438 (11)	0.0568 (13)	0.0330 (10)	0.0028 (9)	0.0154 (9)	0.0018 (9)
C10	0.0445 (12)	0.0769 (17)	0.0438 (12)	−0.0080 (11)	0.0150 (10)	0.0031 (11)
C11	0.0634 (16)	0.0702 (17)	0.0576 (14)	−0.0220 (13)	0.0174 (13)	0.0082 (12)
C12	0.0665 (16)	0.0459 (13)	0.0603 (15)	−0.0044 (11)	0.0137 (13)	−0.0010 (10)
C13	0.0507 (12)	0.0474 (12)	0.0482 (12)	0.0028 (10)	0.0184 (10)	−0.0032 (9)
C14	0.0598 (14)	0.0712 (16)	0.0548 (14)	0.0188 (12)	0.0254 (12)	−0.0006 (11)
C15	0.0616 (17)	0.130 (3)	0.0768 (19)	−0.0217 (17)	0.0397 (15)	−0.0039 (17)
N1	0.0571 (11)	0.0474 (10)	0.0365 (9)	−0.0051 (8)	0.0279 (8)	−0.0030 (7)
O1	0.0729 (11)	0.0630 (10)	0.0374 (8)	−0.0119 (8)	0.0306 (8)	−0.0022 (6)
Cl1	0.1000 (6)	0.0811 (5)	0.1190 (7)	−0.0323 (4)	0.0592 (5)	0.0043 (4)

### Geometric parameters (Å, °)

**Table:**

C1—O1	1.231 (2)	C9—C10	1.401 (3)
C1—N1	1.350 (3)	C9—C14	1.502 (3)
C1—C2	1.493 (3)	C10—C11	1.389 (4)
C2—C7	1.385 (3)	C10—C15	1.510 (3)
C2—C3	1.386 (3)	C11—C12	1.374 (3)
C3—C4	1.374 (3)	C11—H11	0.9300
C3—H3	0.9300	C12—C13	1.379 (3)
C4—C5	1.378 (3)	C12—H12	0.9300
C4—H4	0.9300	C13—H13	0.9300
C5—C6	1.365 (3)	C14—H14A	0.9600
C5—Cl1	1.736 (2)	C14—H14B	0.9600
C6—C7	1.381 (3)	C14—H14C	0.9600
C6—H6	0.9300	C15—H15A	0.9600
C7—H7	0.9300	C15—H15B	0.9600
C8—C13	1.386 (3)	C15—H15C	0.9600
C8—C9	1.393 (3)	N1—H1	0.8600
C8—N1	1.427 (2)		
			
O1—C1—N1	122.86 (18)	C11—C10—C9	119.2 (2)
O1—C1—C2	120.85 (18)	C11—C10—C15	120.0 (2)
N1—C1—C2	116.28 (16)	C9—C10—C15	120.7 (2)
C7—C2—C3	118.69 (19)	C12—C11—C10	121.7 (2)
C7—C2—C1	122.75 (18)	C12—C11—H11	119.1
C3—C2—C1	118.55 (17)	C10—C11—H11	119.1
C4—C3—C2	121.0 (2)	C11—C12—C13	119.5 (2)
C4—C3—H3	119.5	C11—C12—H12	120.2
C2—C3—H3	119.5	C13—C12—H12	120.2
C3—C4—C5	119.0 (2)	C12—C13—C8	119.4 (2)
C3—C4—H4	120.5	C12—C13—H13	120.3
C5—C4—H4	120.5	C8—C13—H13	120.3
C6—C5—C4	121.3 (2)	C9—C14—H14A	109.5
C6—C5—Cl1	118.99 (17)	C9—C14—H14B	109.5
C4—C5—Cl1	119.68 (18)	H14A—C14—H14B	109.5
C5—C6—C7	119.3 (2)	C9—C14—H14C	109.5
C5—C6—H6	120.3	H14A—C14—H14C	109.5
C7—C6—H6	120.3	H14B—C14—H14C	109.5
C6—C7—C2	120.6 (2)	C10—C15—H15A	109.5
C6—C7—H7	119.7	C10—C15—H15B	109.5
C2—C7—H7	119.7	H15A—C15—H15B	109.5
C13—C8—C9	121.75 (19)	C10—C15—H15C	109.5
C13—C8—N1	117.64 (18)	H15A—C15—H15C	109.5
C9—C8—N1	120.59 (18)	H15B—C15—H15C	109.5
C8—C9—C10	118.2 (2)	C1—N1—C8	122.76 (15)
C8—C9—C14	121.53 (19)	C1—N1—H1	118.6
C10—C9—C14	120.3 (2)	C8—N1—H1	118.6
			
O1—C1—C2—C7	157.6 (2)	C13—C8—C9—C14	174.67 (19)
N1—C1—C2—C7	−22.5 (3)	N1—C8—C9—C14	−3.7 (3)
O1—C1—C2—C3	−21.9 (3)	C8—C9—C10—C11	1.9 (3)
N1—C1—C2—C3	157.99 (19)	C14—C9—C10—C11	−177.1 (2)
C7—C2—C3—C4	1.8 (3)	C8—C9—C10—C15	179.5 (2)
C1—C2—C3—C4	−178.6 (2)	C14—C9—C10—C15	0.6 (3)
C2—C3—C4—C5	−0.6 (3)	C9—C10—C11—C12	1.2 (3)
C3—C4—C5—C6	−1.1 (4)	C15—C10—C11—C12	−176.5 (2)
C3—C4—C5—Cl1	178.69 (18)	C10—C11—C12—C13	−2.0 (3)
C4—C5—C6—C7	1.4 (3)	C11—C12—C13—C8	−0.4 (3)
Cl1—C5—C6—C7	−178.32 (17)	C9—C8—C13—C12	3.5 (3)
C5—C6—C7—C2	−0.2 (3)	N1—C8—C13—C12	−178.07 (18)
C3—C2—C7—C6	−1.4 (3)	O1—C1—N1—C8	1.0 (3)
C1—C2—C7—C6	179.04 (19)	C2—C1—N1—C8	−178.89 (17)
C13—C8—C9—C10	−4.2 (3)	C13—C8—N1—C1	117.4 (2)
N1—C8—C9—C10	177.40 (17)	C9—C8—N1—C1	−64.2 (3)

### Hydrogen-bond geometry (Å, °)

**Table:**

Cg1 and Cg2 are the centroids of C2–C7 and C8–C13 rings, respectively.

**Table:**

*D*—H···*A*	*D*—H	H···*A*	*D*···*A*	*D*—H···*A*
N1—H1···O1^i^	0.86	2.18	2.904 (2)	141.
C14—H14A···Cg1^ii^	0.96	2.94	3.653 (2)	132.
C7—H7···Cg2^i^	0.93	2.76	3.612 (2)	154.

Symmetry codes: (i) *x*, −*y*+3/2, *z*+1/2; (ii) *x*+1, *y*, *z*.
